# Entrapment of coronary protection balloon shaft between SAPIEN 3 frames during emergency TAV-in-TAV

**DOI:** 10.1007/s12928-026-01289-9

**Published:** 2026-05-27

**Authors:** Tsuyoshi Kobayashi, Shigeaki Kaga, Kazuhira Omori, Toru Yoshizaki, Kazuto Nakamura, Akira Sato

**Affiliations:** 1 Department of Cardiovascular Medicine, University of Yamanashi, Chuo, Yamanashi Japan; 2 Department of SurgeryⅡ, University of Yamanashi, Chuo, Yamanashi Japan

**Keywords:** TAV-in-TAV, Coronary protection, Balloon shaft entrapment, Stuck leaflet

Acute leaflet immobility (“stuck leaflet”) after balloon-expandable transcatheter aortic valve implantation (TAVI) can cause severe intraprosthetic regurgitation and shock [1]. Here, we report a coronary protection balloon shaft entrapment between prosthesis frames during emergency TAV-in-TAV.

An 83-year-old man underwent transfemoral TAVI with a 23-mm SAPIEN 3 Ultra RESILIA (S3UR). After post-dilation for paravalvular leak, profound hypotension developed (Fig. 1A). Transesophageal echocardiography and aortography revealed torrential aortic regurgitation from a stuck leaflet (Fig. 1B; Video 1).

**Fig. 1:**
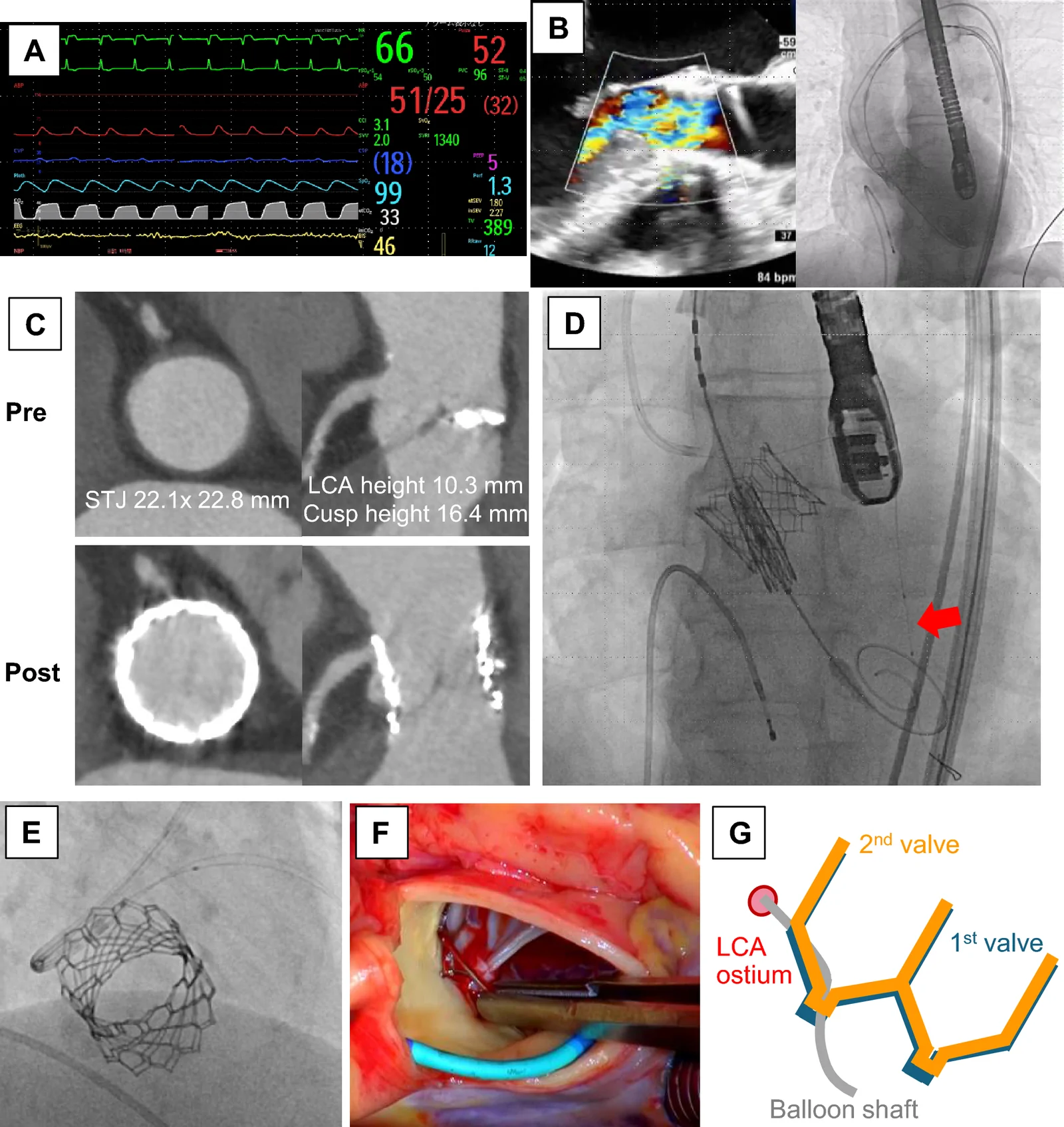
(**A**) Severe hypotension after post-dilation. (**B**) Transesophageal echocardiography and aortography show severe aortic regurgitation. (**C**) CT evaluation of the narrow sinotubular junction and left sinus of Valsalva before and after TAVI. (**D**) Angiography during emergent TAV-in-TAV; red arrow indicates the protection balloon. (**E**) Balloon shaft entrapped between frames. (**F**) Extraction of the jailed shaft with forceps. (**G**) Schema of the jailed shaft

The initial S3UR was implanted at a high level, with a narrow sinotubular junction (STJ) (approximately 22 mm), raising concerns for Valsalva sinus sequestration and the risk of coronary flow obstruction [2] (Fig. 1C). Because a guiding catheter could not pass the STJ from outside the first prosthesis (Fig. 1D), a 3.0-mm noncompliant balloon was advanced over a coronary wire into the left coronary artery via a cell of the first prosthesis for prophylactic protection. A second 23-mm S3UR was deployed within the first prosthesis (Video 2). Hemodynamics stabilized, but the balloon shaft became firmly jailed between the overlapping frames and unretrievable despite repeated balloon inflation/deflation, microcatheter support, and snaring attempts. (Fig. 1E; Video 3). Emergency surgery confirmed that the frames overlapped with the balloon shaft tightly wedged at the top of the frames (Fig. 1F–G, Video 4). The balloon was removed without explanting the transcatheter valve. The patient was discharged home without prosthesis dysfunction or coronary artery compromise.

This report highlights the risk of coronary protection balloon-shaft jailing during urgent TAV-in-TAV in the setting of a narrow STJ. To the best of our knowledge, this is the first reported case of coronary protection balloon-shaft entrapment between overlapping balloon-expandable transcatheter heart valve frames during emergency TAV-in-TAV. Although chimney stenting might have prevented sinus sequestration and possibly reduced the risk of balloon-shaft entrapment by limiting complete overlap of the two valves, the chimney stent itself could have been severely crushed or deformed because it would have been sandwiched between the first and second S3UR valve frames. To mitigate this risk, consider (i) a guide-extension catheter to shield the shaft and (ii) deeper second-prosthesis deployment (more ventricular). Surgical backup should be in place.

## Supplementary Information

Below is the link to the electronic supplementary material.

Supplementary file1 (MP4 22109 kb)

Supplementary file2 (MP4 2240 kb)

Supplementary file3 (MP4 41300 kb)

Supplementary file4 (MP4 7746 kb)
